# PROtective Ventilation with a low versus high Inspiratory Oxygen fraction (PROVIO) and its effects on postoperative pulmonary complications: protocol for a randomized controlled trial

**DOI:** 10.1186/s13063-019-3668-x

**Published:** 2019-11-01

**Authors:** Xue-Fei Li, Dan Jiang, Yu-Lian Jiang, Hong Yu, Jia-Li Jiang, Lei-Lei He, Xiao-Yun Yang, Hai Yu

**Affiliations:** 1 Department of Anesthesiology, West China Hospital, Sichuan University, Chengdu, 610041 Sichuan China; 2 Department of Gynecology, West China Second University Hospital, Sichuan University, Chengdu, 610041 Sichuan China

**Keywords:** Postoperative pulmonary complications, Lung-protective ventilation, Fraction of inspired oxygen, Abdominal surgery

## Abstract

**Background:**

Postoperative pulmonary complications (PPCs) are the most common perioperative complications following surgical site infection (SSI). They prolong the hospital stay and increase health care costs. A lung-protective ventilation strategy is considered better practice in abdominal surgery to prevent PPCs. However, the role of the inspiratory oxygen fraction (FiO_2_) in the strategy remains disputed. Previous trials have focused on reducing SSI by increasing the inhaled oxygen concentration but higher FiO_2_ (80%) was found to be associated with a greater incidence of atelectasis and mortality in recent research. The trial aims at evaluating the effect of different FiO_2_ added to the lung-protective ventilation strategy on the incidence of PPCs during general anesthesia for abdominal surgery.

**Methods and design:**

PROtective Ventilation with a low versus high Inspiratory Oxygen fraction trial (PROVIO) is a single-center, prospective, randomized controlled trial planning to recruit 252 patients undergoing abdominal surgery lasting for at least 2 h. The patients will be randomly assigned to (1) a low-FiO_2_ (30% FiO_2_) group and (2) a high-FiO_2_ (80% FiO_2_) group in the lung-protective ventilation strategy. The primary outcome of the study is the occurrence of PPCs within the postoperative 7 days. Secondary outcomes include the severity grade of PPCs, the occurrence of postoperative extrapulmonary complications and all-cause mortality within the postoperative 7 and 30 days.

**Discussion:**

The PROVIO trial assesses the effect of low versus high FiO_2_ added to a lung-protective ventilation strategy on PPCs for abdominal surgery patients and the results should provide practical approaches to intraoperative oxygen management.

**Trial registration:**

www.ChiCTR.org.cn, identifier: ChiCTR18 00014901. Registered on 13 February 2018.

**Electronic supplementary material:**

The online version of this article (10.1186/s13063-019-3668-x) contains supplementary material, which is available to authorized users.

## Background

About 2.0 to 5.6% of more than 234 million patients undergoing surgery develop postoperative pulmonary complications (PPCs), especially after general and vascular surgeries (approximately 40%), which makes PPCs the most common perioperative complications following surgical site infection (SSI) [1–6]. PPCs, especially respiratory failure, add to the morbidity and mortality risk in hospitalized patients [1, 4, 5]. Moreover, PPCs prolong hospital stay and increase medical expense and resource utilization [2, 5]. A reduction of PPCs is a very important evaluation index of medical quality management. A possible explanation for increasing morbidity in patients who develop PPCs is that mechanical ventilation under general anesthesia results in gas-exchange impairment, a local inflammatory response and circulatory disorder [7, 8]. Thus, decreased lung volumes, ventilator-induced lung injury and atelectasis are strongly associated with the incidence of PPCs [9].

Prior studies noted that so-called lung-protective ventilation, referring to low-tidal-volume (V_T_), appropriate positive end-expiratory pressure (PEEP) level and recruitment maneuvers, seems to be the optimum option for the surgical and intensive care unit (ICU) populations [10–13]. The decrease in PPCs, mortality and health care costs have been observed in the protective-ventilated population. On the basis of the robust evidence available, a combination of low V_T_ (6–8 ml/kg of predicted body weight) [11, 14], a level of PEEP at 5–8 cmH_2_O [15] and repeated recruitment maneuvers [16] are now widely adopted.

Setting the inspiratory oxygen fraction (FiO_2_) intraoperatively is a significant task of anesthetists, but has not been based on evidence-based guidelines. Obtaining comprehensive knowledge about hyperoxia caused by high FiO_2_ has been stressed as important by clinicians over the past few decades, including its potentially deleterious effects on lung. Even mildly elevated FiO_2_ levels have been reported to exacerbate lung injury by up-regulating pro-inflammatory cytokines and inducing neutrophil infiltration in the alveolar spaces [17–19].

Even if there is no significant difference in pulse oximetry and the oxygenation index for several time-points with 30 or 80% FiO_2_ intraoperatively, hyperoxia and substantial oxygen exposure are common in clinical practice [20, 21]. Questions have been raised about the use of oxygen in ventilated patients undergoing elective surgery. A recent systematic review revealed that the trials of this decade about the effects of FiO_2_ on SSI have been inconclusive, and we should also focus on clinically relevant pulmonary side-effects and other adverse events (AEs) [22–25]. In addition, exposure to oxygen is related to adverse effects in critically ill patients [26, 27]. The ideal FiO_2_ level in the lung-protective ventilation strategy to protect against PPCs and improve clinical outcomes has not been addressed in the perioperative period.

The relationship between FiO_2_ and PPCs in surgical patients is mainly affected by a hyperoxia-induced change in the respiratory mechanism. Higher FiO_2_ seems to be associated with pulmonary complications and adverse clinical outcomes, but the existing evidence is insufficient to warrant its effect to promote PPCs [28–30]. We hypothesize that a low level of FiO_2_ (30%) compared with high FiO_2_ (80%) could decrease the incidence of PPCs in patients undergoing abdominal surgery when lung-protective ventilation strategy is administered.

## Methods and design

### Study design

The PROVIO trial is a single-center, prospective, randomized controlled and two-arm study and is conducted in accordance with the Declaration of Helsinki. The trial will be conducted in West China Hospital of Sichuan University, China. We aim to assess the effect of FiO_2_ in a lung-protective ventilation strategy, in an abdominal surgical population of patients, on PPCs, extrapulmonary complications (e.g., SSI, sepsis, etc.), hospital stay and mortality.

**Fig. 1:**
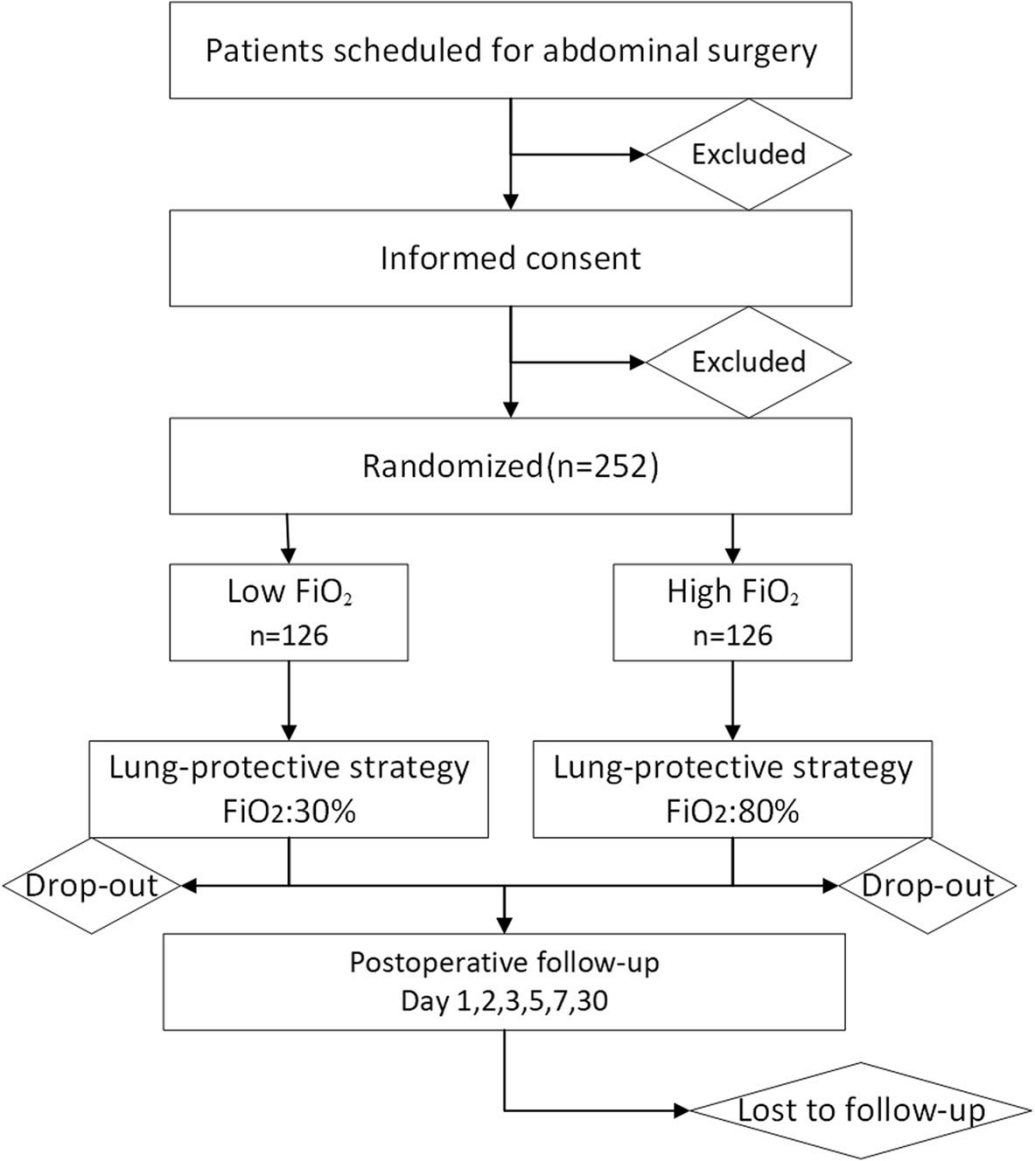
Consolidated Standards of Reporting Trials (CONSORT) diagram of the PROVIO trial

The protocol follows the Standard Protocol Items: Recommendations for Interventional Trials (SPIRIT) 2013 Statement. The SPIRIT checklist can be found inAdditional file 1. The diagram of the Consolidated Standards of Reporting Trials (CONSORT), which are also followed, is presented in Fig. 1.

### Study population

The inclusion criteria of the study are: American Society of Anesthesiologists (ASA) physical status I–III patients aged 18 years or older, scheduled for elective abdominal surgery with an expected duration of at least 2 h and planned to be extubated in the operating room. Laparotomy and laparoscopy surgery will not be restricted. Patients are ineligible if they have suffered from pneumothorax, acute lung injury or acute respiratory distress syndrome within the last 3 months. Other exclusion criteria are: a diagnosis of heart failure (New York Heart Association classes; NYHA IV), chronic renal failure (glomerular filtration rate < 30 ml/min), serious hepatic diseases (e.g., hepatic failure), scheduled for reoperation or postoperative mechanical circulatory support, known pregnancy, participation in another interventional study, and with a body mass index (BMI) of > 30 kg/m^2^.

### Randomization, blinding and bias minimization

Patients will be recruited from West China Hospital of Sichuan University. Consecutive male or female patients aged 18 years or older who will undergo abdominal surgery under general anesthesia are screened for study eligibility. Randomization will be performed using a computer-generated randomization list (SPSS 22.0) with an allocation rate of 1:1. The allocation is concealed in an opaque envelope and will be sent to the attending anesthetist by a blinded investigator.

Given the characteristics of the study, the attending anesthetist must know the intervention. Researchers, including the investigator in the operating room, the data collector and the data analyzer, will all be blinded to the randomization arm. All the surgeons, nurses and anesthetists in the post-anesthesia care unit (PACU) will not know the allocation. Postoperative visits and outcome assessment will be performed by a blinded investigator. Emergency unblinding is permissible if hypoxemia occurs (defined as pulse oxygen saturation (SpO_2_) < 92% or partial pressure of oxygen in arterial blood (PaO_2_) < 60 mmHg).

### Standard procedures

The risk of PPCs will be assessed using the Assess Respiratory Risk in Surgical Patients in Catalonia (ARISCAT) risk score [31] before randomization (Table 1). An investigator assesses the individual risk of PPCs with the seven predictors of the ARISCAT risk score (age, SpO_2_, respiratory infection in the last month, preoperative anemia, duration of surgery, and whether an emergency procedure). The ARISCAT score will help to analyze the effect of FiO_2_ to intermediate-high-risk patients who obtain a score of more than 26. All patients receiving an assessment will be included and randomized.

**Table 1:** Assess Respiratory Risk in Surgical Patients in Catalonia (ARISCAT) risk score in the logistic regression model

	βcoefficient	Score *
Age (years)		
≤ 50	0	0
51–80	0.331	3
> 80	1.619	16
Preoperative SpO_2_ (%)		
≥ 96	0	0
91–95	0.802	8
≤ 90	2.375	24
Respiratory infection in the last month		
No	0	0
Yes	1.698	17
Preoperative anemia (Hb ≤10 g/dl)		
No	0	0
Yes	1.105	11
Surgical incision		
Peripheral	1.480	15
Upper abdominal	2.431	24
Intrathoracic		
Duration of surgery (h)		
≤ 2	1.593	0
2–3	2.268	16
> 3	0.768	23
Emergency procedure		
No	0	0
Yes	0.768	8

*A risk score ≥ 26 predicts an intermediate to high risk for postoperative pulmonary complications after abdominal surgery. The simplified risk score was the sum of each logistic regression coefficient multiplied by 10, after rounding off its value

*Hb* hemoglobin

All randomized participants will receive standard care and monitoring including a five-lead electrocardiogram, SpO_2_, blood pressure (invasive or noninvasive) and end-tidal carbon dioxide (E_T_CO_2_). The attending anesthetist responsible for the patient can choose the bispectral index (BIS), muscle-relaxant monitoring and cardiac-output monitoring techniques according to clinical routines.

There will be no restriction to the anesthetic regimen and individualized health care will be performed intraoperatively. Use of antiemetics and muscle-relaxant antagonists (mainly neostigmine) will be recorded in the case report form (CRF).

### Intraoperative ventilatory management

Pre-oxygenation will be prescribed for 5 min at 100% FiO_2_ using a mask. In accordance with the group allocation, the participants will be randomized to receive a low (30%) or a high (80%) FiO_2_ throughout the whole period of intraoperative mechanical ventilation after tracheal intubation. The FiO_2_ level is implemented through adjusting the air-oxygen ratio when the total gas flow remains at 2 L/min. The FiO_2_ in our protocol refers to the actual fraction of inspired oxygen presented in the display panel on the anesthesthetic machine. Table 2 shows the ventilation settings.

**Table 2:** Intraoperative ventilation settings for the PROVIO trial

	Low-FiO_2_ group	High-FiO_2_ group
FiO_2_	0.30	0.80
V_T_	8 ml/kg	8 ml/kg
PEEP	6–8 cmH_2_O	6–8 cmH_2_O
I:E	1:2	1:2
RR	Adjusted according to E_T_CO_2_ (35–45 mmHg)	Adjusted according to E_T_CO_2_ (35–45 mmHg)
P_max_	30 cmH_2_O	30 cmH_2_O

*E*_*T*_*CO*_*2*_ end-tidal carbon dioxide, *FiO*_*2*_ inspiratory oxygen fraction, *I:R* Inspiratory to Expiratory ratio, *PEEP* positive end-expiratory pressure, *P*_*max*_ maximum peak airway pressure, *RR* respiratory rate, *V*_*T*_ tidal volume

Intraoperative ventilation in all participants will be performed via the lung-protective ventilation strategy. A recruitment maneuver with peak airway pressure (P_aw_) of 30 cmH_2_O for 30 s will be performed instantly after intubation, every 60 min after intubation and before extubation. Other settings are shown in Table 2. Ventilatory parameters, including tidal volume (V_T_), minute volume (MV), airway pressure (P_aw_), plateau pressure (P_plat_), fresh gas flow, PEEP and FiO_2_, will be monitored.

After extubation, patients will be sent to the PACU or the ward where they will be oxygenated with 2 L/min, pure oxygen via a nasal tube over 24 h. At the same time, they will receive standard monitoring.

### Intraoperative care

After induction, standard intraoperative care will be applied in both groups to reach a target of standard state (Table 3). Vasoactive drugs can be used in patients with unstable hemodynamics as appropriate.

**Table 3:** Standard state target

	Parameter	Value
Hemodynamics	Mean arterial pressure (MAP)	70 mmHg < MAP < 100 mmHg
Hemodynamics	Heart rate (HR)	50/min < HR < 100/min
Oxygenation	Pulse oxygen saturation (SpO_2_)	≥ 92%

### Rescue strategies for intraoperative hypoxemia

Around 30% FiO_2_ has been proved to be safe in mechanically ventilated patients and rarely causes hypoxemia [21]. We designed a rescue strategy for patients in whom SpO_2_, measured by pulse oximetry, fell to less than 92% or PaO_2_ to less than 60 mmHg for more than 1 min.

Endotracheal-tube displacement, airway-secretion blockage, bronchospasm, pneumothorax, and hemodynamic change would all be checked for. After excluding these as underlying causes, a rescue recruitment maneuver with P_aw_ 30 cmH_2_O for 30 s will be implemented [12, 16, 32]. If this were to fail, FiO_2_ and ventilation settings would be altered until acquiring the required oxygenation (SpO_2_ ≥ 92% or PaO_2_ ≥ 60 mmHg).

### Outcome measurements

The primary outcome is the occurrence of pulmonary complications within the first 7 days postoperatively. The definition of PPCs follows the ARISCAT study (respiratory infection, respiratory failure, bronchospasm, atelectasis, pleural effusion, pneumothorax or aspiration pneumonitis.) [4].

The secondary outcomes include the occurrence of PPCs in the postoperative 30 days; SSI, postoperative nausea and vomiting (PONV) in the postoperative 7 days; the severity grade of pulmonary complications in the postoperative 7 and 30 days (Table 4); and death rate in the postoperative 7 and 30 days.

**Table 4:** Grades of pulmonary complications

Postoperative pulmonary complication grade	
Grade 1	Cough, dry Microatelectasis: abnormal lung findings and temperature > 37.5 °C without other documented cause; normal chest radiograph Dyspnea, not due to other documented cause
Grade 2^a^	Cough, productive, not due to other documented cause Bronchospasm: new wheezing or preexistent wheezing resulting in a change in therapy Hypoxemia: SpO_2_ < 90 in room air Atelectasis: gross radiological confirmation (concordance of 2 independent experts) plus either temperature > 37.5 °C or abnormal lung findings Hypercarbia (PaCO_2_ > 50 mmHg), requiring treatment
Grade 3	Pleural effusion, resulting in thoracentesis Pneumonia: radiological evidence (concordance of 2 independent experts) plus clinical symptoms (two of the following: leukcocytosis or leukopenia, abnormal temperature, purulent secretions), plus either a pathological organism (by Gram stain or culture), or a required change in antibiotics Pneumothorax Noninvasive ventilation, strictly applied to those with all of the following: (a) SpO_2_ ≤ 92% under supplemental oxygen; (b) need of supplemental oxygen > 5 L/min; and (c) respiratory rate ≥ 30 bpm Reintubation postoperative or intubation, period of ventilator dependence does not exceed 48 h
Grade 4	Ventilatory failure: postoperative ventilator dependence exceeding 48 h, or reintubation with subsequent period of ventilator dependence exceeding 48 h
Grade 5	Death

^a^We only classified as grade 2 if two or more items in grade 2 were present

*PaCO*_*2*_ partial pressure of arterial carbon dioxide, *SpO*_2_ pulse oxygen saturation

Pulmonary complications will be scored with a grade scale ranging from 0 to 5 adapted from Kroenke et al., Hulzebos et al., Fernandez-Bustamante et al. and Canet et al. [4, 5, 33, 34]. Grade 0 in the scale represents no PPCs, grades 1–4 represent increasing severity levels of pulmonary complications, and grade 5 represents death before discharge. SSI will be defined with the criteria from the Centers for Disease Control and Prevention (CDC) [35].

Tertiary outcomes in the first 7 and 30 days postoperatively are as follows:

1. Sepsis: the infection-centric systemic response which needs to meet two or more criteria of the systemic inflammatory response syndrome (SIRS) [36]
2. Septic shock: defined as a composite of sepsis-induced response, perfusion abnormalities, and hypotension despite adequate fluid resuscitation [36]
3. Myocardial ischemia [37]
4. Heart failure [37]
5. Urinary system infection [37]

Acute kidney injury: defined according to the Kidney Disease Improving Global Outcomes (KDIGO) criteria [38]

6. Anastomotic fistula
7. Reintubation
8. Unplanned admission to the ICU
9. Hospital length of stay postoperatively

### Data collection and follow-up

**Fig. 2:**
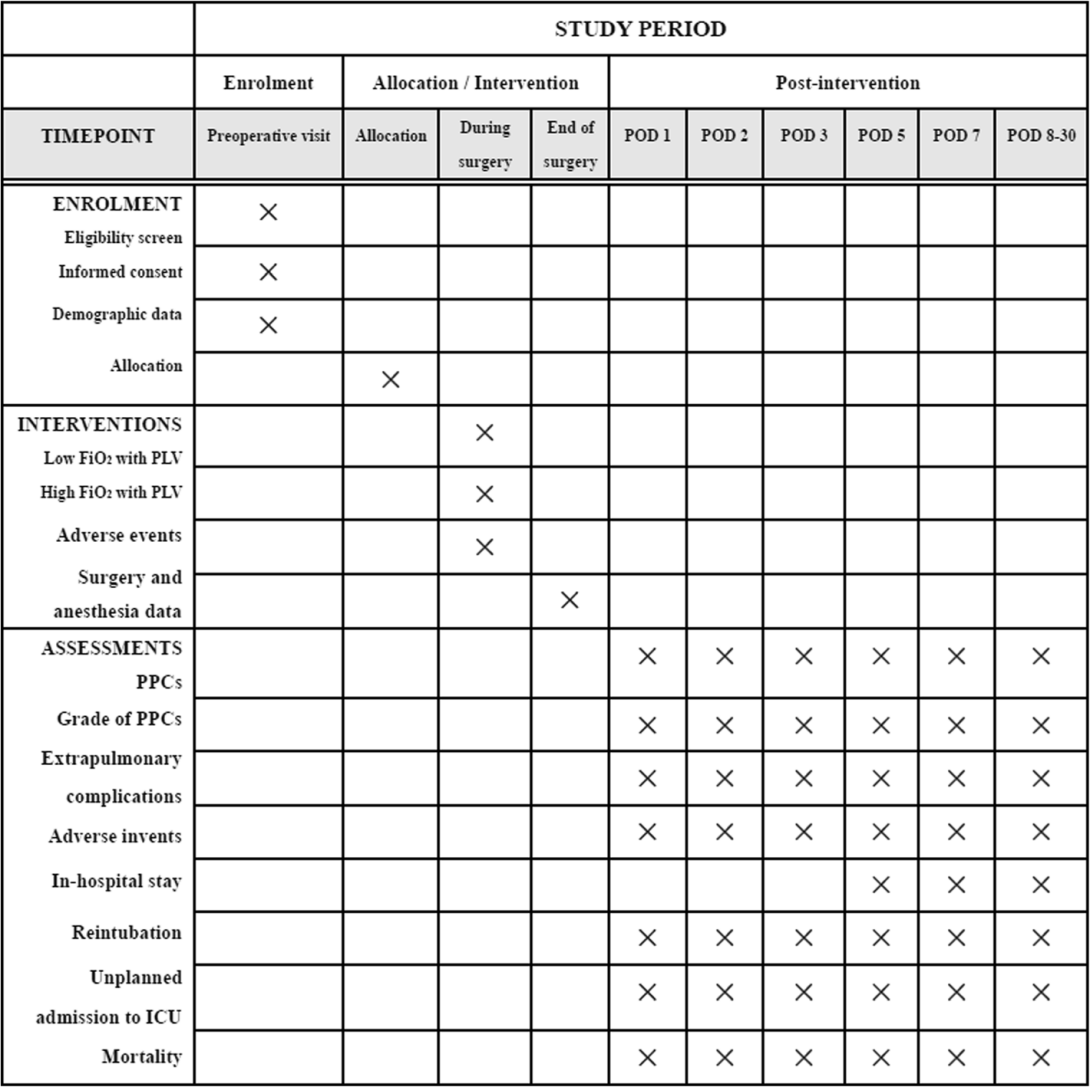
Standard Protocol Items: Recommendation for Interventional Trials (SPIRIT) schedule of enrollment, interventions and assessments

The study will be conducted in the operating room and visits are restricted during the screening, hospitalization and follow-up periods. The primary and secondary outcomes will be measured on postoperative days 1, 2, 3, 5 and 7 or at discharge by interview. On postoperative day 30, participants will be contacted by phone (Fig. 2). Demographic and baseline data will be collected preoperatively, which include age, sex, weight, BMI, ASA physical status, ARISCAT risk score, smoking status, pulmonary status (chronic pbstructive pulmonary disease (COPD), atelectasis, asthma respiratory infection within the last 3 months, use of ventilatory support), routine laboratory tests (hemoglobin, white blood cell count, platelet count, neutrophil count) and medical history.

Both intraoperative surgery- and anesthesia-associated data will be recorded, including type of surgery, surgical incision or approach, duration of surgery and ventilation, blood loss, transfusion of blood products, fluid balance (calculated by subtracting the measurable fluid losses from measurable fluid intake during anesthesia), drugs during anesthesia (e.g., anesthetics and antiemetics), adjustment of ventilatory parameters or FiO_2_, hypoxemia events, the need for rescue strategy, number of emergency recruitment maneuvers, and unplanned admission to the ICU.

Postoperative visits will be conducted daily and clinical data required to assess PPC grade include body temperature, lung auscultation, symptoms (e.g., cough, expectoration and dyspnea), chest-imaging manifestations, and laboratory tests. Surgical incision assessment, PONV and other outcomes will also be measured and collected daily according to the evaluation criterion mentioned above.

The Data and Safety Monitoring Board (DSMB) composed of five independent individuals is set up to supervise the overall conduct of the study (the screening, recruitment and adherence to the protocol). The DSMB is responsible for checking and ensuring the completeness and validity of data recording. The interim analysis will be conducted when the first 120 participants are recruited and have been visited to completion. The DSMB has access to patient allocation, but the results of the interim analysis will be treated as strictly confidential.

### Study drop-out

Participants have the right to withdraw from the study at any time without any consequences for further treatment. Investigators have the right to terminate the study at any time in consideration of the best interests of the participants. Both situations will be recorded in the CRF and discussed.

Any AEs and treatment side-effects will be sent to the DSMB which will discussed whether the participant should drop out accordingly.

### Statistical considerations

The sample size required was estimated based on the investigative data in our medical center. The pilot study showed that PPCs (respiratory infection, respiratory failure, bronchospasm, atelectasis, pleural effusion, pneumothorax or aspiration pneumonitis) occurred in 50.4% patients received 80% FiO_2_ after abdominal surgery (sample size: 100). And assuming a round 50% rate of PPCs in the high-FiO_2_ (80%) group, we calculated that a total sample size of 252 patients (126 in each group) will have 80% power to detect a relative risk reduction of 35% in PPCs between groups, at a two-sided alpha level of 0.05 and 5% drop-out. We will conduct a sample size reassessment after recruiting half of the patients for safety consideration.

All statistics will be analyzed by SPSS 22.0 statistical software (IBM Corporation, Chicago, IL, USA) through the intention-to-treat principle, which covers all randomized patients receiving surgery. Participants with adjusted FiO_2_ values are still treated as the low-FiO_2_ population when analyzed. In a descriptive analysis of the population, mean and standard deviation (SD) will be used for normally distributed variables, medians and interquartile ranges used for non-normally distributed variables and percentages used for categorical variables. Stratified description will be used as appropriate.

There will be a baseline comparison of age, gender, BMI, type of surgery, surgical approach, duration of surgery and ARISCAT score between groups and logistic regression analysis will be performed if an imbalance between groups exists. The Student’s *t* test will be used for continuous, normally distributed variables and the Mann-Whitney *U* test will be used for continuous, non-normally distributed data. The primary and secondary outcomes will be compared using the *χ*^2^ test or Fisher’s exact test, while multiple logistic-regression analysis will be used to identify hazards. A two-sided *P* value < 0.05 is considered statistically significant.

A custom-made folder is made to store the participants’ data, which consists of documents and forms. Only blinded researchers have access to the folder. Only when the study is complete will the investigators obtain the data.

## Discussion

The optimal intraoperative FiO_2_ remains highly debated. Many physicians consider excessive oxygen supplement a salutary practice which is now widely applied in routine practice due to its simplicity and ease of availability [39]. Despite the controversy, the majority of published randomized trials comparing 30 and 80% FiO_2_, mainly in SSI and PONV, show that intraoperative high FiO_2_ decreases the risk of both [40–42]. Furthermore, new World Health Organization (WHO) recommendations on intraoperative and postoperative measures for SSI prevention in 2016 suggest that patients undergoing general anesthesia with endotracheal intubation for surgical procedures should receive 80% FiO_2_ intraoperatively [43]. What remains controversial is whether the intraoperative use of an elevated FiO_2_ is essential to all intubated patients without hypoxemia, although both 30 and 80% FiO_2_ provide similar oxygenation [21]. A multicenter observational trial collecting the ventilator data 1 h after induction showed that most ventilated patients (83%) in Japan were exposed to potentially preventable hyperoxia, especially in one-lung ventilation patients and the elderly [20].

The “benefit” of this pervasive liberal oxygen management has recently been questioned. Concerns on potential detrimental effects, such as impairing lung-capillary endothelial function and facilitating oxidative stress due to the use of high FiO_2_, were raised [44–46]. Endothelial activation may initiate progressive hyperoxic lung injury when persistently ventilated under hyperoxic conditions at 70% FiO_2_ [19]. In addition, excessive oxygen can lead to pulmonary endothelial-cell damage through mitochondrial fragmentation [47]. This can be explained by the formation of reactive oxygen species (ROS) and pro-inflammatory cytokines in endothelial cells which were found in an animal study [19, 46]. Romagnoli et al. demonstrated that protective ventilation with the lowest level of FiO_2_ to keep the SpO_2_ ≥ 95% reduces oxygen toxicity by generating less ROS production [45]. However, there is a contradictory view on the detrimental effects of high FiO_2_ on endothelial function in healthy volunteers [48]. Another interpretation is that high FiO_2_ may change pulmonary-gas exchange in surgical patients. Ventilation with high FiO_2_ (80–100%) increases the intrapulmonary shunt [49] and impairs gas exchange [50]. In addition, resorption atelectasis results from a phenomenon in which nitrogen is displaced by oxygen which can diffuse more rapidly into the blood. Resorption atelectasis can also promote pulmonary shunting and cause hypoxemia [51]. Ventilation for induction of anesthesia with 100% FiO_2_ leads to significantly larger atelectatic areas than with 60% FiO_2_ [52]. Atelectatic areas tend towards having a low ventilation/perfusion ratio. Hyperoxia is also an important factor contributing to the apoptosis of alveolar epithelial cells and lowers the level of surfactant proteins that indicate damage of the lung tissue [53]. The synergetic action of the above factors increases the risk of lung injury and pulmonary complications.

Indeed, supplemental oxygen results in hyperoxia, and is reported as an independent risk factor for ventilator-associated pneumonia in one observational study [54]. Liberal oxygen use is considered detrimental in mechanically ventilated patients in the aspect of lung function [55] and clinical outcomes [27]. The PROXI trial demonstrated that the incidence of PPCs, PONV and SSI after abdominal surgery were not significantly different in patients receiving 80 or 30% FiO_2_ [56]; nevertheless, the former suffers higher long-term mortality (23.3 vs 18.3%) [57]. Also one observational trial has suggested a dose-dependent manner in FiO_2_ and 30-day mortality. The incidence of PPCs has declined by half in the low-FiO_2_ group with a median of 31% (range 16–34%) [30].

Yet, no direct evidence has revealed the relationship between FiO_2_ in lung-protective ventilation and PPCs, and existing data reported that postoperative pulmonary function is better protected with a relative low FiO_2_ intraoperatively [58]. One systematic review showed that the included trials only focused on postoperative atelectasis, rather than on all forms of PPC [59]. Despite the PROXI trial demonstrating that PPCs did not differ after inhalation of 80 vs 30% oxygen, the results are worth discussing. The emergency surgery population was not excluded in the PROXI trial, emergency surgery being an independent risk factor for pulmonary complications [4]. Intubation time is also a key element for causing pneumonia and atelectasis. Moreover, the complication measures of PROXI lacked a standard and comprehensive judgment evaluation, which only assessed the three types of PPC (atelectasis, pneumonia and respiratory failure) according to the CDC criteria. And, above all, the ventilation strategy for patients is not specified, which plays a key role in the incidence of pulmonary complications. The iPROVE-O_2_ trial is an ongoing randomized controlled trial (ClinicalTrials.gov identifier: NCT02776046) comparing the efficacy of 80 and 30% FiO_2_ using an individualized, open-lung ventilatory strategy in reducing the incidence of SSI [60]. The major differences compared to the PROVIO trial are: the appearance of pulmonary complications as one of the secondary outcomes; individualized, open-lung ventilation as the ventilatory mode which is a combination of 8 ml/kg V_T_, recruitment maneuver and the optimal individualized PEEP. The recruitment maneuver will be performed by a PEEP-titration trial [61]. Undoubtedly, the individualized, open-lung ventilation strategy is more complex to implement clinically when compared to lung-protective ventilation [61].

The limitations of our study must be mentioned. Firstly, we conducted a pilot study to determine the incidence of PPCs in our medical center with reference to the sample size calculation. We hope that our results will provide the possible direction and reference for subsequent research into FiO_2_. Secondly, the study excludes the patients scheduled for some types of surgery because of the duration of the surgery. Thirdly, the oxygenation index and arterial oxygen pressure that may reflect the actual oxygenation state will not be measured during the perioperative period.

In the absence of an intraoperative lung-protective ventilation strategy, previous studies failed to identify a certain relationship between FiO_2_ and PPCs. We insist that lung-protective ventilation in both groups will reduce bias regarding ventilation-associated impact, and enhance lung protection. Conclusively, the PROVIO trial is the first clinical trial focusing on the effects of FiO_2_ added to lung-protective ventilation on PPCs. The results of the trial should support anesthetists in routine oxygen management during general anesthesia in an attempt to prevent PPCs.

## Trial status

The trial is ongoing from February 2018, and expected to complete in May 2019. The protocol version is 3.0 (issue date: 25 December 2018).

## Additional file

Additional file 1: Standard Protocol Items: Recommendations for Interventional Trials (SPIRIT) Checklist for the protocol of a clinical trial. (DOCX 25 kb)

## Data Availability

The datasets used and/or analyzed during the current study are available from the corresponding author on reasonable request.

## Abbreviations

AE: Adverse event
ARISCAT: Assess Respiratory Risk in Surgical Patients in Catalonia
ASA: American Society of Anesthesiologists
BIS: The bispectral index
BMI: Body mass index
CDC: The Centers for Disease Control and Prevention
CONSORT: Consolidated Standards of Reporting Trials
COPD: Chronic obstructive pulmonary disease
CRF: Case report form
DSMB: Data and Safety Monitoring Board
E_T_CO_2_: End-tidal carbon dioxide
FiO_2_: Inspiratory oxygen fraction
Hb: Hemoglobin
HR: Heart rate
I:E: Inspiratory to Expiratory ratio
ICU: Intensive care unit
KDIGO: Kidney Disease Improving Global Outcomes
MAP: Mean arterial pressure
MV: Minute volume
NYHA: New York Heart Association classes
P_plat_: Plateau pressure
PACU: Post-anesthesia care unit
PEEP: Positive end-expiratory pressure
PONV: Postoperative nausea and vomiting
PPCs: Postoperative pulmonary complications
PROVIO: PROtective Ventilation with a low versus high Inspiratory Oxygen fraction trial
ROS: Reactive oxygen species
SD: Standard deviation
SIRS: Systemic inflammatory response syndrome
SPIRIT: Standard Protocol Items: Recommendations for Interventional Trials
SpO_2_: Pulse oxygen saturation
SSI: Surgical site infection
V_T_: Tidal volume
